# Data presenting a modified bacterial expression vector for expressing and purifying Nus solubility-tagged proteins

**DOI:** 10.1016/j.dib.2016.07.032

**Published:** 2016-07-21

**Authors:** Nidhi Gupta, Heng Wu, Jonathan R. Terman

**Affiliations:** Departments of Neuroscience and Pharmacology, Harold C. Simmons Comprehensive Cancer Center, The University of Texas Southwestern Medical Center, Dallas, TX 75390, USA

**Keywords:** Mical, Plexin, Semaphorin, Repulsion, Axon guidance, Redox

## Abstract

Bacteria are the predominant source for producing recombinant proteins but while many exogenous proteins are expressed, only a fraction of those are soluble. We have found that a new actin regulatory enzyme Mical is poorly soluble when expressed in bacteria but the use of a Nus fusion protein tag greatly increases its solubility. However, available vectors containing a Nus tag have been engineered in a way that hinders the separation of target proteins from the Nus tag during protein purification. We have now used recombinant DNA approaches to overcome these issues and reengineer a Nus solubility tag-containing bacterial expression vector. The data herein present a modified bacterial expression vector useful for expressing proteins fused to the Nus solubility tag and separating such target proteins from the Nus tag during protein purification.

**Specifications Table**

**Table:** 

Subject area	*Molecular Biology*
More specific subject area	*Recombinant protein expression in bacteria*
Type of data	*Figure*
How data was acquired	*Molecular biology and recombinant DNA approaches*
Data format	*Formatted*
Experimental factors	*Standard genetic engineering and bacterial approaches were employed*
Experimental features	*Modified bacterial expression vector*
Data source location	*Departments of Neuroscience and Pharmacology**,** Harold C. Simmons Comprehensive Cancer Center**,** The University of Texas Southwestern Medical Center**,** Dallas, TX 75390**,** USA*
Data accessibility	*Data is with this article*

**Value of the data**

• The data provide a Nus protein fusion partner (tag) vector to increase the solubility of target proteins expressed in bacteria.
• The data will allow for the ease of use of affinity purification-mediated separation of a target protein from the Nus tag.
• The data will support the use of the Nus solubility tag in bacteria for target protein expression, solubility, and purification.

## 1 Data

**Fig. 1:**
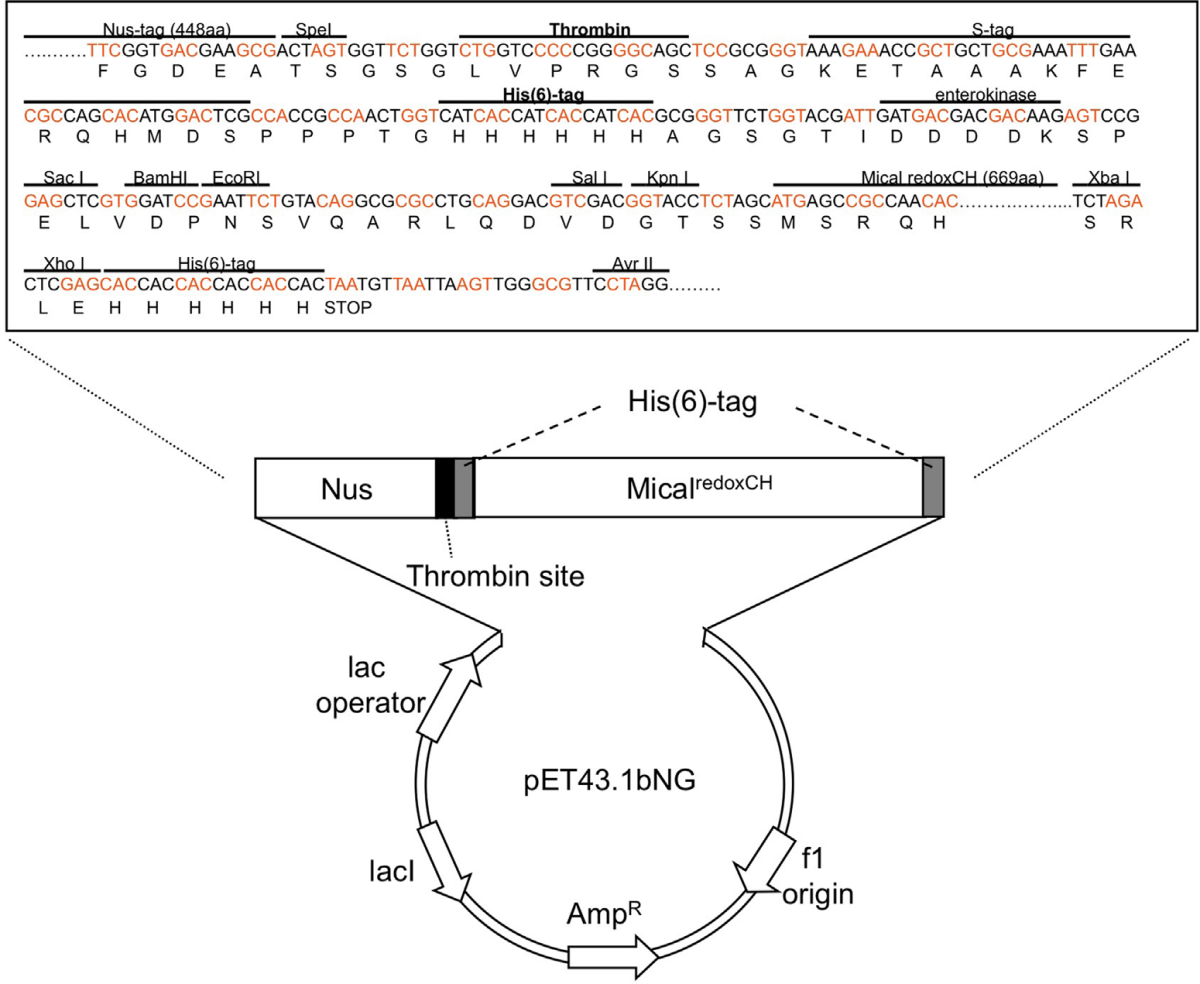
pET43.1bNG – a modified bacterial expression vector with a Nus solubility tag for expressing and purifying proteins such as the Mical^redoxCH^ protein.

We engineered a Nus solubility tag-containing bacterial expression vector for expressing Nus solubility-tagged proteins and then removing the Nus tag from the target protein during protein purification (Fig. 1). We have called this vector pET43.1bNG (Fig. 1), and this configuration allows separation of target proteins from the Nus tag following thrombin digestion and His/Nickel affinity chromatography. The data presented also allowed us to use a Nus solubility tag to increase the solubility of Mical protein and then separate and purify His-tagged Mical protein from the Nus tag [1].

## 2 Experimental design, materials and methods

### 2.1 Bacterial culture and transformation

Standard approaches for culturing bacterial cells were employed [2]. In particular, 50 μl of competent bacteria (Electromax DH10B cells; Life Technologies) was added into a 1.7 ml microcentrifuge tube. In order to amplify the pET43.1 vector, ~1 μg of pET43.1b plasmid (Novagen) was then added into the microcentrifuge tube containing the competent bacteria. The plasmid and competent bacteria were then incubated on ice for 30 min. Competent bacteria were then heat shocked for 1 min at 42 °C to allow uptake of the plasmid into the bacteria. The tube was then placed on ice for 2 min. 950 μl of liquid SOC medium was then added to the transformation mixture and incubated for 1 h at 37 °C in a shaking incubator (~200 rpm). 100 μl of the transformed bacteria was then spread onto luria broth (LB) agar 10 cm plates containing 50 μg/ml carbenicillin and the plates were incubated ~14–16 h at 37 °C to allow bacterial colonies to grow. A single clone from the culture plate was then selected using sterile technique [2], [3] and inoculated into 2 ml of LB culture medium containing 50 μg/ml carbenicillin and shaken at 37 °C overnight in a shaking incubator. The overnight culture was then prepared to isolate the pET43.1b plasmid DNA using standard approaches [2].

### 2.2 Design and molecular biology to generate pET43.1bNG

The configuration of the pET43.1 Nus- and His-tag containing vector prevented thrombin or enterokinase-mediated separation of His-tagged target proteins from the Nus-tag, since the vector contained His-tags on both sides of these protease sites. Therefore, we modified the pET43.1b vector to interchange the position of the thrombin cleavage site and the N-terminal His tag. Following thrombin digestion, this new configuration allowed us to separate the target protein from the Nus tag using His/Ni^2+^ affinity chromatography [1]. To do this, we designed primers containing a 5′ Spe I restriction enzyme site (Forward: 5′– GAC GAA GCG ACT AGT GGT TCT GGT CTG GTC CCC CGG GGC AGC TCC GCG GGT AAA GAA ACC GCT GCT GCG –3′) and a 3″ PshA I restriction enzyme site (Reverse: 5′– TCC CGG ACT CTT GTC GTC GTC ATC AAT CGT ACC AGA ACC CGC GTG ATG GTG ATG GTG ATG ACC AGT TGG CGG TGG CGA GTC CAT GTG –3′) and modified sequence based on switching the position of the thrombin cleavage site and the N-terminal His tag. We then used PCR to amplify the short fragment and after digestion of the appropriately sized PCR product with Spe I and PshA I, the fragment was inserted into the Spe I (ACTAGT) and PshA I (GACAAGAGTC) sites of the pET43.1b vector. Ligation of DNA and transformation of bacteria was then performed using standard approaches as described above. The transformed bacteria was then spread onto LB agar 10 cm plates containing 50 μg/ml carbenicillin and the plates were incubated ~14–16 h at 37 °C to allow bacterial colonies to grow. Single clones from the culture plate were then selected using sterile technique as described above and each clone was inoculated into separate 2 ml tubes of LB culture medium containing 50 μg/ml carbenicillin and shaken at 37 °C overnight in a shaking incubator. The overnight culture was then prepared to isolate the pET43.1bNG plasmid DNA using standard approaches as described above, restriction enzyme digested to confirm the correct product, and sequenced on both strands.

### 2.3 Design and molecular biology to generate Mical^red^^o^^xCH^ pET43.1bNG

Our work with Mical has revealed that the oxidoreductase (redox) and calponin homology (CH) domains of Mical [4], [5] are together sufficient for Mical׳s effects on actin filaments in vivo and in vitro with purified proteins [6], [7], [8]. Therefore, we have concentrated on working with Mical protein containing both the redox and CH domains (Mical^redoxCH^). To generate Mical protein containing the redox and CH domains (Mical^redoxCH^) in pET43.1bNG, a portion of the Mical protein containing the CH domain was PCR amplified using primers containing a 5′ Xho I restriction enzyme site (Forward: 5′–TGA CAG TCT GCT CGA GCC GTT TTG GCC CAC TGG ATC –3′) and 3′ Avr II restriction enzyme site (Reverse: 5′ – GTT TTA TCA GCC TAG GAA CGC CCA ACT TAA TTA ACA TTA GTG GTGGTG GTGGTG GTG CTC GAG TCT AGA GCG AAA CAA GTC GCA GAT CTG GTC C –3′) and after digestion of the appropriately sized PCR product with Xho I and Avr II, the fragment was inserted into the Xho I and Avr II sites of a previously generated pET43.1bNG vector containing the redox domain of Mical. This placed the CH domain of Mical, which we had previously found to be necessary in vivo for Mical׳s function [6], in frame with the redox domain. Ligation of DNA and transformation of bacteria was then performed using standard approaches as described above. The transformed bacteria was then spread onto LB agar 10 cm plates containing 50 μg/ml carbenicillin and the plates were incubated ~14–16 h at 37 °C to allow bacterial colonies to grow. Single clones from the culture plate were then selected using sterile technique as described above and each clone was inoculated into separate 2 ml tubes of LB culture medium containing 50 μg/ml carbenicillin and shaken at 37 °C overnight in a shaking incubator. The overnight culture was then prepped to isolate the Mical^redoxCH^ pET43.1bNG plasmid DNA using standard approaches as described above, digested to confirm the correct product, and sequenced on both strands.
